# A Measurement of ‘Walking-the-Wall’ Dynamics: An Observational Study Using Accelerometry and Sensors to Quantify Risk Associated with Vertical Wall Impact Attenuation in Trampoline Parks

**DOI:** 10.3390/s21217337

**Published:** 2021-11-04

**Authors:** Imam Hossain, Shilei Zhou, Karlos Ishac, Edward Lind, Lisa Sharwood, David Eager

**Affiliations:** 1 Faculty of Engineering and Information Technology, University of Technology Sydney, Sydney 2007, Australia; MDImam.Hossain@uts.edu.au (I.H.); Karlos.Ishac@uts.edu.au (K.I.); edward.lind@student.uts.edu.au (E.L.); David.Eager@uts.edu.au (D.E.); 2 Faculty of Medicine and Health, University of Sydney, Sydney 2006, Australia; lisa.sharwood@sydney.edu.au

**Keywords:** trampoline parks, trampolinist, trampoline safety, trampoline manoeuvre, parkour, taekwondo, injury risk, accelerometer, stability

## Abstract

This study illustrates the application of a tri-axial accelerometer and gyroscope sensor device on a trampolinist performing the walking-the-wall manoeuvre on a high-performance trampoline to determine the performer dynamic conditions. This research found that rigid vertical walls would allow the trampolinist to obtain greater control and retain spatial awareness at greater levels than what is achievable on non-rigid vertical walls. With a non-rigid padded wall, the reaction force from the wall can be considered a variable force that is not constrained, and would not always provide the feedback that the trampolinist needs to maintain the balance with each climb up the wall and fall from height. This research postulates that unattenuated vertical walls are safer than attenuated vertical walls for walking-the-wall manoeuvres within trampoline park facilities. This is because non-rigid walls would provide higher g-force reaction feedback from the wall, which would reduce the trampolinist’s control and stability. This was verified by measuring g-force on a horizontal rigid surface versus a non-rigid surface, where the g-force feedback was 27% higher for the non-rigid surface. Control and stability are both critical while performing the complex walking-the-wall manoeuvre. The trampolinist experienced a very high peak g-force, with a maximum g-force of approximately 11.5 g at the bottom of the jump cycle. It was concluded that applying impact attenuation padding to vertical walls used for walking-the-wall and similar activities would increase the likelihood of injury; therefore, padding of these vertical surfaces is not recommended.

## 1. Introduction

The trampoline was invented by George Nissen [1] and was used during World War II to train pilots by getting them used to orientating themselves in the air. In more recent times, NASA has used trampolines to prepare astronauts for space travel. Trampoline usage simulates the feeling of weightlessness and prepares astronauts for zero gravity [2]. Trampolines were first included in the 2000 Olympics Games in Sydney, Australia [3]. Trampolines offer people of all ages the fun and excitement of experiencing zero gravity in their backyard on a domestic-grade trampoline, by visiting their local trampoline facility, or by joining a gymnastics association and seeking formal training in the skill of trampolining.

**Figure 1:**
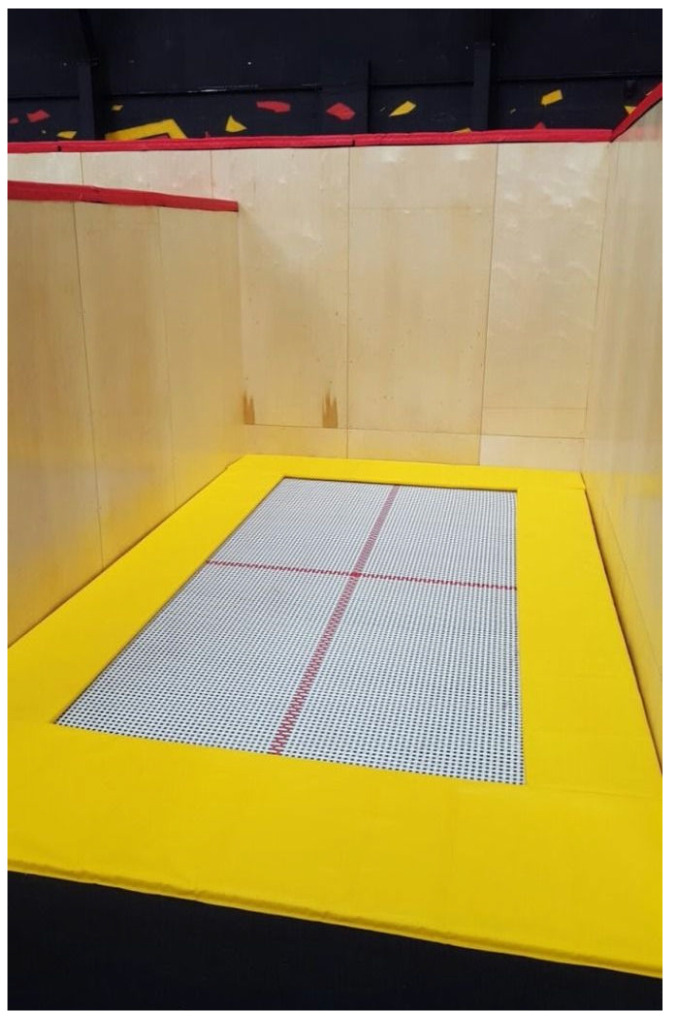
High-performance trampoline with three walls located within the free space of the trampoline.

Walking-the-wall is a manoeuvre seen in the indoor trampoline park environment, where an experienced trampolinist walks or runs up a vertical wall that is located in the free space of the trampoline (see Figure 1). The walking-the-wall is a challenging manoeuvre and, for safety reasons, should only be attempted by highly skilled trampolinists.

If you have ever had the pleasure of attending a Circus du Soleil performance, you will have witnessed the magic of people seemingly defying gravity as they walk up vertical walls. These circus performers provide us with an excellent example of the walking-the-wall manoeuvre.

Before attempting the walking-the-wall manoeuvre, participants must be physically fit and already experienced trampolinists. They must be proficient in bouncing and falling to land on their back. This manoeuvre may sound easy, but is counter-intuitive upon first attempt. It can be practised on the side of a trampoline, where the participant can simply lean backward and practise falling onto their back, putting their chest forward as they return to the vertical position, always pivoting on their feet. Self-propulsion of the chest cavity assists with forward motion as the trampolinist returns to a vertical position. The learned technique of raising one’s chest forward is important as it allows a progressively higher wall position with each successive bounce. Another helpful technique in the overall skill of walking-the-wall is kicking the legs in contrary motion as the back leaves the mat. This requires abdominal muscle control while the back is horizontal with the bed, the upper legs are bent vertically from the hip, and the lower legs are horizontal to the bed. Mastery of these techniques in coordination is required before the walking manoeuvre can be introduced, the body driving up and forward toward the wall. The walking will commence with the dominant leg forward, and as the trampolinist touches the wall at the point of reaching the height in each bounce, the chest is lifted. Increasing the stride length will give additional height, as will raising the arms toward the ceiling upon impacting the bed, ready to bounce up again. The trampolinist uses their lower leg to lift them up the wall balance and the upper leg for balance. Importantly, when the trampolinist reaches the top-of-cycle, they kick out and push themselves toward the trampoline bed so as to increase their potential energy and gain extra height. Conversely, small strides result in a loss of balance. Mastery of these skills drives performance and height attainment, such that with each successive bounce, the top of the wall is attained.

When the trampolinist lands on their back, this serves to increase the fall height and potential energy relative to landing on the legs. The increased potential energy is equal to m×g×Δh, where Δh is the trampolinist’s leg height measured from the base of their foot to their bottom.

Walking-the-wall can be performed without a trampoline. An example of this is parkour, or free running, where the practitioners use their momentum by running towards a vertical surface, onto which they continue running in upward motion, achieving similarly to the trampolinist, as they move around complex environments [4]. The point of difference here is that the parkour master does not fall backward onto their back to continue the motion.

Although trampolining is a high-risk activity that can cause serious injuries, there are many physical health benefits if practised safely. Measurement and tracking methods have also improved as a result of Olympic scoring requirements and academic research explorations. This allows us to more deeply understand the biomechanics involved in trampolining, which is of use to high-performance trampolinists wishing to optimise their technique and precision, however, which may also lead to improved safety.

There are various methods used to analyse the motions and biomechanics of trampolinists. The methods differ according to the purpose of observation. For example, an Olympic trampoline scoring system will differ from a scientific evaluation setup. Research conducted in [5] evaluated the use of force plates to measure trampolining performance. This differs from the conventional laser system used in Olympic grading. The study observed some time shift delays due to the high elasticity of the trampoline, but improved on the conventional system due to the force plates’ abilities to detect the location of the athlete. Another study by [6] used video tracking to analyse the kinematics and energy of trampolinists at a popular amusement park. More recently, the combination of computer vision and AI-based algorithms has become increasingly popular for pose estimation. For example, Connolly et al. [7] used computer vision techniques and a convolutional neural network to estimate the pose of a trampoline athlete’s body in real time. The body orientation and joint angles could be estimated automatically with 80.7% accuracy.

The research conducted by [8] observed the gaze of trampolinists while performing a somersault. The results found that all subjects fixed their gaze on a specific point on the trampoline bed to prepare for landing and that their eyes moved continuously downwards to counteract the backwards head movement. Furthermore, it was observed that gymnasts of higher skill levels fixated on the bed at a later time point in their descent than less experienced athletes. A similar study performed in [9] measured the gaze and head and eye movements during somersaults with full twists on the trampoline. A portable eye-tracking device and motion capture suit was used to evaluate the results. Similarly to [8], it was observed that gymnasts use the trampoline bed as a fixation point for orientation and more skilled athletes fixate at a significantly later stage.

In recent years, the tracking of trampolinists’ motion and behaviours has been combined with digital media to create unique and immersive experiences. In a system presented by [10], the user jumping on the trampoline is tracked by an RGB-D camera, which is used to play a game. In the game, the user must jump up various platforms. The results showed that most users preferred the game-enabled training as opposed to self-training. A system presented in [11] developed a multiplayer version of a similar game, where the trampolinists compete against each other. The jumping position of the trampolinist is tracked and projected in a 2D plane in the game and displayed to both users in real time. The objective is to jump on top of your opponent. The authors concluded that exer-games (exercise combined with games) provided a means of empowerment for the users that could lead to increased motivation towards physical activity.

This paper discusses the novel use of vibration equipment to assess the forces experienced by a trampolinist performing the walking-the-wall manoeuvre on a high-performance trampoline. In our proposed system, we implemented a tri-axial accelerometer and gyroscope sensors on the trampolinist performing the walking-the-wall manoeuvre in order to evaluate the kinematics and biomechanics involved in the trampolinist as well as the influence of surfaces in the trampoline setup. Cameras were used to record the trampolinist’s manoeuvre. The findings of this paper help to understand the force interactions between the trampolinist and the wall, which could be used to improve the safety of trampoline performance by correctly manoeuvring and contacting the wall and trampoline. This research also provides evidence for the claim that unattenuated vertical walls used for walking-the-wall activities in trampoline park facilities are safer than attenuated vertical walls [12].

## 2. Benefits and Risks Associated with Trampolining

Trampolining has many benefits for physical health and strength training. A study conducted by [13] compared two groups of children, where one group undertook a 12-week trampolining training course and the other group did no physical exercise. It was observed that the trampolining group demonstrated increased static balance, dynamic balance, and vertical jump at the end of the program. Another study presented in [14] evaluated the use of a mini-trampoline as a rehabilitation device for balance training for those with ankle instability. The study compared two groups that undertook a 6-week training course using either a mini-trampoline or a Dura-disc. The results showed that there were improvements in postural sway for both groups and that the mini-trampoline was an effective balance training device after lateral ankle sprain. Extensively, another study presented in [15] compared the effects of trampoline training on balance, gait, and fall efficacy for stroke patients. The study also separated the patients into two groups, with one group undergoing additional trampoline-based training. The studies demonstrated that trampoline training resulted in significantly improved balance, dynamic gait, and fall efficacy of stroke patients compared to the non-trampoline-trained group. The research conducted in [16] analysed the effect of trampoline training on children with autism spectrum disorder (ASD) through a 20-week training program. Children with ASD tend to have lower engine performance. The results showed that the trampoline course improved the motor proficiency in the children, especially in bilateral coordination, balance, speed, upper body coordination, and strength.

The use of trampolining has clear benefits to improving balance, gait, and postural sway as aforementioned. We have also analysed human biomechanics and posture in recent years, as well as the effect of interventions as a means for physical training [17,18]. In addition to these benefits, researchers in more recent years have focused their attempts on forming more direct links between trampolining and its effects on performance in other sports. A study in [19] specifically links trampoline training to improving taekwondo technique performance. With all the benefits in mind, it is crucial that any practitioner follows safety guidelines to prevent injuries from trampolining.

The practice of trampolining incorporates many benefits; however, this activity also incorporates risk. These risks become a matter of concern among the general public, in the event of serious and injurious incidents. There are international examples of individuals suffering life-altering disability as a result of such incidents. Risk-related injury can occur in all sporting activities and can range from mild to severe, very occasionally resulting in death. On the positive side, trampolining exercises can strengthen every muscle, organ, and cell in the body. Trampolining is not only fun but will enhance overall coordination, strength, flexibility, proprioception, timing, balance, cardiovascular fitness, lymphatic circulation, rhythm, bilateral motor skills, and bone density. On the negative side, trampolining can result in serious injuries due to high g-force acceleration, including spinal injuries, which may result in the trampolinist spending the rest of their life in a wheelchair. For instance, the g-force experienced during a 48 km/h front-end car crash is 30 g [20]. Backyard trampolines are a source of fun and exercise for children, but can also carry a risk of serious injury [21,22,23,24,25,26,27]. Trampoline park facilities offer a greater range of activities but they too can be a source of serious injury [28,29].

Trampoline-related injuries are well documented in prior research and are a matter of consideration for all related studies [30,31,32]. In recent years, researchers have documented the patterns of trampoline-related injuries in order to understand the risks involved and improve the safety of the device for future use. In a study conducted in Norway [30], the results showed that 77% of injuries occurred on the trampoline frame and 22% of injuries occurred by falling off the trampoline. The study also described that around three quarters of all injuries involved more than one person on the trampoline simultaneously. This observation was also seen by [32]. In [30], it was also concluded that trampolining is a high-risk activity and can cause serious injuries in the neck and elbow areas in young children, emphasising that trampolines should still be used under proper safety guidelines.

The study conducted by [31] observed marked differences in injury epidemiology between trampoline parks and home trampolines. Overall, it was found that sprains and fractures were the most common types of injuries in both settings. However, trampoline parks resulted in more lower-body injuries compared to home trampoline installations. Furthermore, home trampoline installations resulted in a higher number of head injury occurrences. Another study conducted by [32] investigated patterns in trampoline-related injuries among inpatients at a children’s hospital in Ireland. Among study subjects, the average age of injury was 8.5 years and the main injuries were fractures and soft tissue injuries. The investigation showed that 57% of patients were on the trampoline with another person and that having more than one person on the trampoline posed a greater risk of injury, where the lighter person is 14-times more likely to be injured than the heavier person.

## 3. Trampolinist Manoeuvre Recording Methods

To analyse the trampolinist’s manoeuvre during trampoline activities, this research measured the accelerations and velocities, as well as the body postures, of the trampolinist. Only data from one trampolinist were collected. This trampolinist was selected as a person who was proficient in both walking-the-wall and jumping activities on a high-performance trampoline. This trampolinist’s height was 1.75 m and mass was 78 kg. Two separate but identical high-performance trampoline beds capable of providing more than 10 g were used for the walking-the-wall and jumping activities.

For the acceleration and velocity measurement, Camomilla et al. [33] published a systematic review on trends supporting the in-field use of wearable inertial sensors for sports performance. Roerll et al. [34] noted that it was important that there was little to no relative movement between the sensor and the athlete. This lack of relative movement was achieved in this study by mounting the sensor in a tight pocket within a purpose-built bra-like compression garment, which held the portable inertial measurement unit (IMU) in position during all data acquisition. In this way, we aligned with the best practice recommendation to locate the IMU close to the centre-of-mass of the trampolinist where possible.

In this study, all dynamic measurements were taken with the Yost Labs 28-gram tri-axial attitude and heading reference system device [35] mounted against the sternum of the trampolinist. This device has additional capabilities, such as on-board orientation processing and data filtering, compared to a standard IMU device. The device was configured for capturing both accelerometer and gyroscope sensor outputs and also retrieved the orientation of the player using a Kalman filter. The device produced corrected readings from both the accelerometer and gyroscope sensors in g-force and rad/s units, respectively. The sampling frequency was 54 Hz, which was automatically determined by the device for this particular activity. The full-scale deflection of the accelerometer was ±24 g, and for the gyroscope, the full-scale deflection was ±2000 °/s. The orientation of the activity was processed by the device in quaternion form, as is preferable for preserving the correct orientation without gimbal lock issues. Similar IMU devices have been used by other researchers [36,37,38].

**Figure 2:**
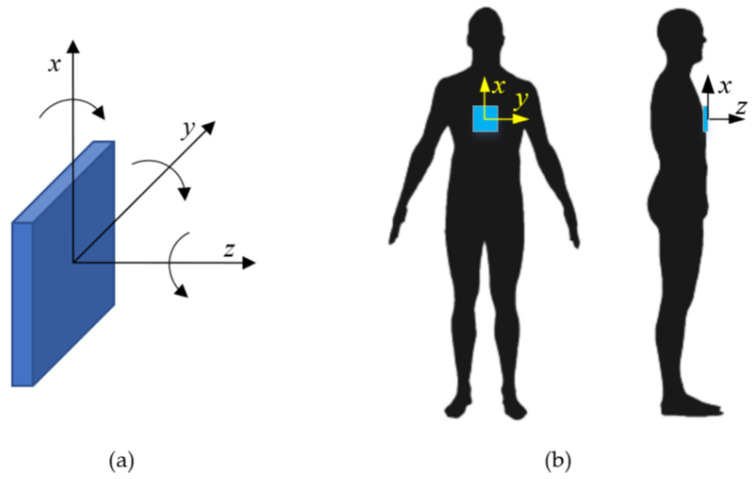
(**a**) Yost Labs tri-axial device *x*–*y*–*z* coordinate orientation. (**b**) The device was mounted close to the sternum of the trampolinist.

Figure 2a depicts the IMU device’s initial co-ordinate system orientation. Figure 2b depicts the location of the IMU device relative to the trampolinist, where the IMU was installed close to the trampolinist’s chest:

- *x*-direction indicates the vertical motion, where up and clockwise rotation is positive;
- *y*-direction indicates the side-wise motion, where left and anti-clockwise rotation is positive; and
- *z*-direction indicates the depth motion (towards the vertical wall), where forward and anti-clockwise rotation is positive.

As can be seen from Figure 2a, the IMU co-ordinate system setup did not follow the standard convention but, rather, was configured to be compatible with computer 3D modelling and visualisation software. This was to ensure that IMU data analysis could be carried out efficiently using computer software, without needing data transformation. Initial measurements were also conducted with the sensor mounted on the back of the trampolinist. These measurements proved to be unreliable due to the levels of shock noise within the signal generated each time the trampolinist’s back impacted with the trampoline bed, sandwiching the sensors between the body and trampoline mat.

To record the trampolinist’s body posture during the trampoline activities, two Sony DSC RX10 M3 digital cameras capable of high-frame-rate (HFR) shooting were positioned at 90° to obtain both the front and side view of the activity. The video capture rate was set at 50 and 500 frames per second for standard and HFR shooting, respectively.

## 4. Trampolinist Manoeuvre Recording Results and Analysis

While the IMU was mounted, the trampolinist repeatedly conducted the walking-the-wall and jumping motions. During this period, the trampolinist’s activities were recorded by the IMU and the digital cameras.

### 4.1. Walking-the-Wall Manoeuvre Recording and Analysis

**Figure 3:**
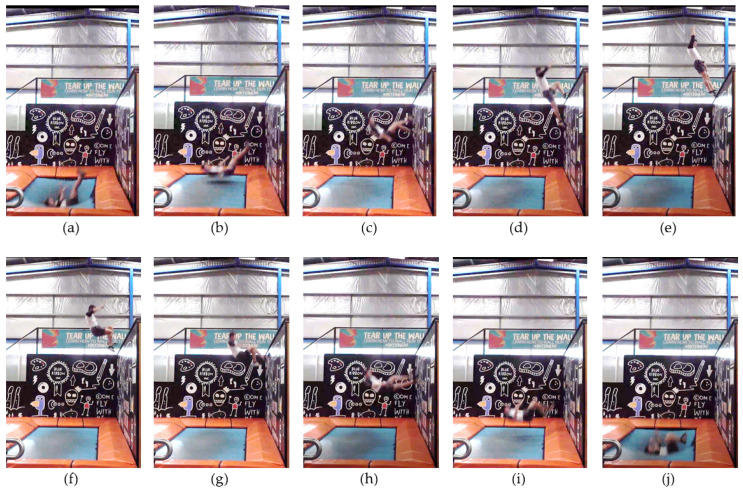
Walking-the-wall using a high-performance trampoline—side view of the trampolinist.

One complete cycle during the walking-the-wall sequence is shown in Figure 3. It starts from when the trampolinist bounces up from the trampoline bed and ends when the trampolinist reaches the bottom-dead-centre on the trampoline bed.

Figure 3a depicts the trampolinist at the bottom-dead-centre location and the reference for the commencement of a complete cycle. Figure 3b depicts the trampolinist’s motion before touching the wall. The trampolinist can be seen stretching his legs toward the wall to assist in elevating his upper body. Figure 3c depicts the trampolinist walking the wall. Figure 3d depicts the trampolinist as his upper foot reached the top of the wall. Figure 3e depicts the trampolinist with both feet on the nose of the ledge at the top of the wall. Figure 3f depicts the trampolinist as he commences the decent from the top of the wall. Figure 3g depicts the trampolinist as he continues his decent walking down the wall. Figure 3h depicts the trampolinist immediately prior to him pushing out and down. Figure 3i depicts the trampolinist as he pushes out and down. Figure 3j depicts the trampolinist falling from the wall and landing on the trampoline again. Note that the trampolinist’s legs are bent at the hips and knees and his arms are pointing toward the ceiling. His back is evenly distributed on the bed.

Figure 3a–e depict the trampolinist’s ascending process from the bottom of the trampoline to the top of the wall. It can be seen that the trampolinist’s body posture changes from lying on his back with his legs higher than his torso to nearly standing upright. During this process, strong lumbar abdomen and leg strength and balance ability are required for the trampolinist. Moreover, the wall also plays an important role in performing this activity. The trampolinist always needs to tread the wall and use the wall reaction force to adjust his body, which means that the wall should be hard enough to help the trampolinist to obtain an adequate solid grasp on the wall.

During the descending process shown in Figure 3f–j, less effort from the trampolinist is required on adjusting his body posture and maintaining his balance. However, when the trampolinist is pushing out and down, it still requires a hard wall to provide a large reaction force so that the trampolinist can obtain a higher falling speed and move deeper into the trampoline to obtain the desired bounce force for the next walking-the-wall cycle.

**Figure 4:**
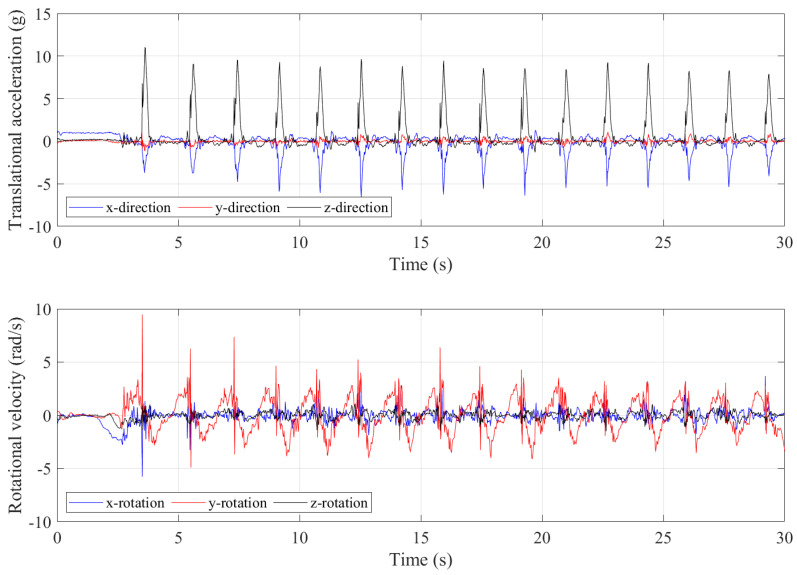
Translational accelerations and rotational velocities during walking–the–wall.

**Figure 5:**
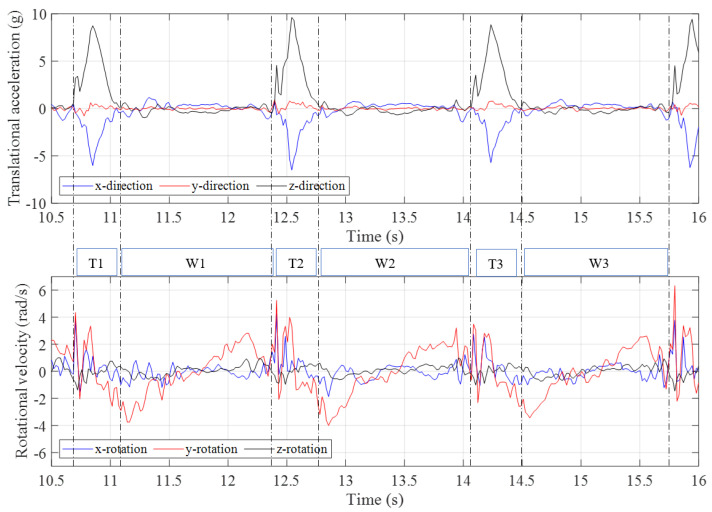
Three complete manoeuvre cycles of translational accelerations and rotational velocities during walking–the–wall.

Approximately 30 s of data were collected with the trampolinist walking the wall. The g-force accelerations and and angular velocities in different axes relative to the IMU device’s local inertial frame of reference are shown in Figure 4. As can be seen from the plot, each of the high peaks related to the event closely coincide with the dynamic condition of the trampolinist in the position shown in Figure 3a. Figure 5, shows three walking-the-wall cycles under dynamic conditions as extracted from the IMU device. In Figure 5, T_*x*_ represents the contact with the trampoline bed phase and W_*x*_ represents the walking-the-wall phase.

It can be seen that that the g-force acceleration in the *y*-direction is significantly lower compared with the *x*– and *z*–directions. The angular velocities around the *x*–axis and *z*-axis are considerably lower compared with the angular velocity around the *y*–axis. These results indicate that the trampolinist’s motion was predominantly in the *x*–*z* plane and his lateral movement, lateral bend, and twist were minor during walking-the-wall, which is also in accord with the visual observations.

The peaks in the g-forces of the *x*-direction and *z*-direction indicated that the trampolinist reached the bottom-dead-centre on the trampoline bed. At this moment, the trampoline had the largest deformation and provided the largest force to the trampolinist. At the bottom-dead-centre, the trampolinist lay on the trampoline with his back. Therefore, the positive g-force acceleration in the *z*-direction represented the reaction acceleration of the trampolinist moving upwards and the negative g-force acceleration in the *x*-direction represented the reaction acceleration of moving towards the wall.

**Figure 6:**
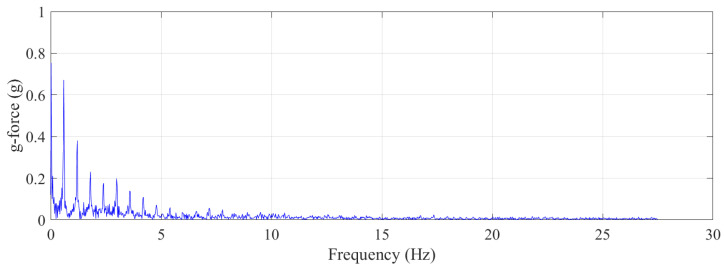
Trampolinist walking-the-wall activity frequency component breakdown using fast Fourier transform algorithm.

Figure 6 shows frequency component analysis using fast Fourier transform (FFT) in a walking-the-wall activity cycle for *x*-direction g-force data, as this was the dominant axis. As can be seen from the plot, there were 10 significant frequency components in the walking-the-wall activity, which can be decomposed into namely 0.022, 0.59, 1.21, 1.80, 2.39, 2.99, 3.58, 4.2, 4.79, and 5.4 Hz. The second frequency component, 0.59 Hz, is closely related to a single foot contacting the wall; the third frequency component, 1.21 Hz, is closely related to both feet contacting the wall, and the fourth frequency component, 1.80 Hz, is closely related to the trampolinist bouncing off the trampoline bed. It can be said that, for a trampolinist who is experienced in the activity, there are 10 frequency components to be expected for a non-injurious activity.

### 4.2. Jumping Manoeuvre Recording and Analysis

**Figure 7:**
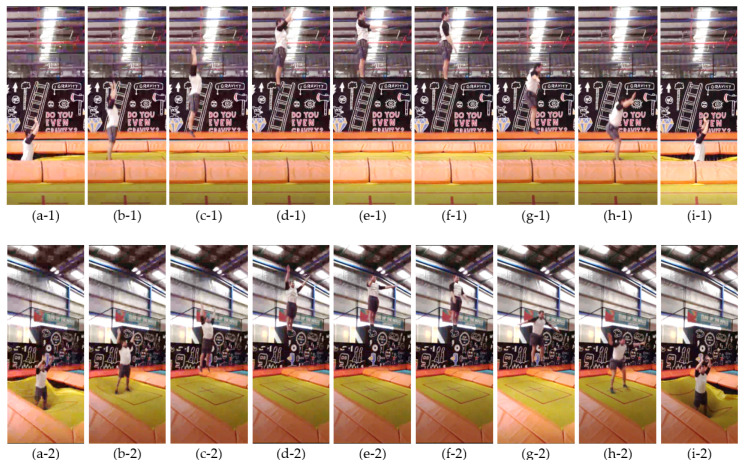
Jumping using a high-performance trampoline. (**Top**) Side view of the trampolinist. (**Bottom**) Front right view of the trampolinist.

A separate activity purely consisting of jumping on the trampoline was also carried out besides the walking-the-wall activity. One complete cycle of jumping is shown in Figure 7, in which the trampolinist’s motion at each selected instant is demonstrated by both the side view (indicated by −1) and front right view (indicated by −2). Figure 7a–d depict the ascending process and Figure 7f–i depict the descending process, while Figure 7e depicts the trampolinist’s posture at the highest point.

At the beginning of the jumping motion, as shown in Figure 7a–c, the trampolinist had to stretch his arms to adjust his body and achieve a balance in the air.

When the trampolinist was in the air and with certain height above the trampoline, as shown in Figure 7d–f, he was in a balanced, stable, and free state. He could perform different movements, such as bending or twisting his body and legs.

During the landing process, as shown in Figure 7g–i, the trampolinist had to split his legs and stretch his arms to obtain balance control when he touched the trampoline. Moreover, he also needed to slightly bend his legs for landing buffering and reinforcing the next jump. From the above analysis, it could be found that the skills in body balancing, posture control, and reaction force control play an important role in safely and correctly conducting walking-the-wall and jumping motion. Thus, this motion analysis provides a valuable reference for trampoline training and learning by decomposing the motions.

**Figure 8:**
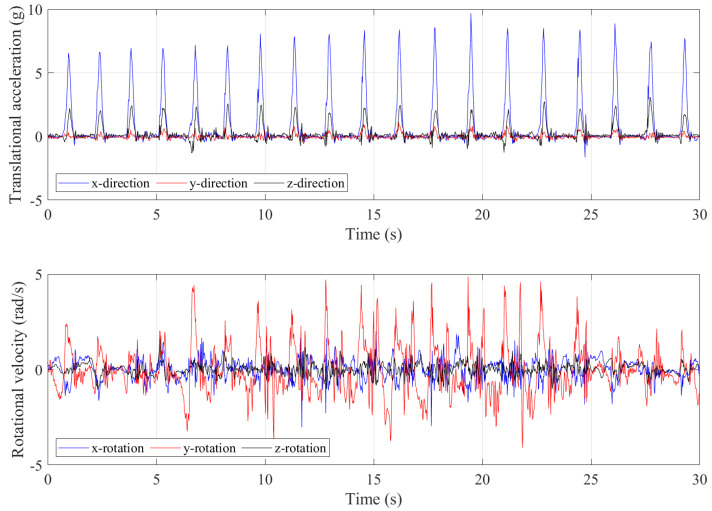
Translational accelerations and rotational velocities during jumping.

Approximately 30 s of data were collected with the trampolinist during jumping. The g-force accelerations and angular velocities are shown in Figure 8. Although this was a repetitive action of jumping by the trampolinist, the buildup of the trampolinist’s momentum was evident from the g-force peaks.

**Figure 9:**
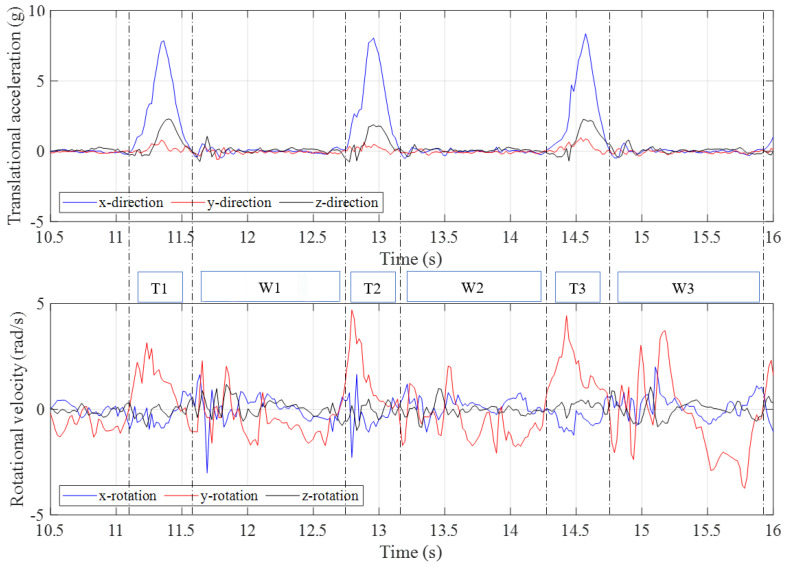
Three complete manoeuvre cycles of translational accelerations and rotational velocities during jumping.

Figure 9 shows three complete manoeuvre cycles as extracted from the IMU device. In Figure 9, T*x* represents the *contact with the trampoline bed* phase and W*x* represents the in the air phase.

The peaks in the accelerations of the *x*-direction and *z*-direction indicate that the trampolinist reached the bottom-dead-centre on the trampoline bed. At the bottom-dead-centre, the positive acceleration in the *x*-direction represents the reaction g-force on the trampolinist for pushing the body upwards. Ideally, in the jumping motion, the trampolinist is only moving up and down so there should be g-force only in the *x*-direction. However, as shown in Figure 7g–i, the trampolinist bent his legs for buffering and balancing during landing. Thus, he did not perfectly stand up straight on the trampoline. The body inclination introduces a acceleration component in the *z*-direction. This was also evident from the angular velocity data of the *y*-axis where the values were abrupt and frequently reached a peak. Similar to walking the wall, the lateral acceleration was quite small, which means that the trampolinist maintained less lateral movements or side bending.

**Figure 10:**
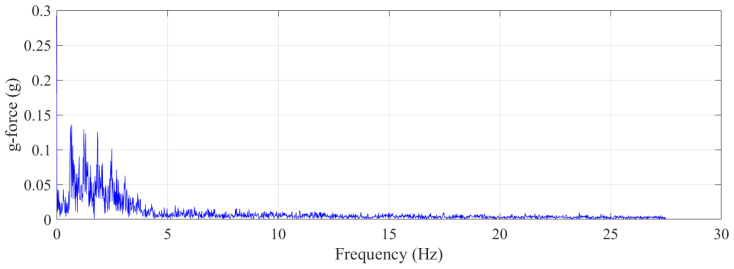
Trampolinist jumping activity frequency component breakdown using fast Fourier transform algorithm.

Figure 10 shows frequency component analysis using FFT in a jumping activity cycle for *z*-direction g-force data, as this was the dominant axis for the balancing act. As can be seen from the plot, there were seven significant frequency components in the jumping activity, which can be decomposed into namely 0.67, 1.22, 1.84, 2.49, 3.08, 3.65, and 4.28 Hz. The third frequency component, 1.84 Hz, is closely related to a single foot contacting the wall; the third frequency component, 1.21 Hz, is closely related to the trampolinist bouncing off the trampoline bed. Finally, it can be said that the jumping activity required less balancing compared to the walking-the-wall activity as there were less significant frequency components and the required g-force in the components was considerably less.

## 5. Trampolinist Force Safety Discussion

Safety is of paramount importance during any trampolining activity. It is even more important in the design of high-performance activities such as walking-the-wall as the window for error is narrower.

The force acting on the trampolinist’s body should be investigated to ensure that these forces are maintained below known safety threshold levels. The force safety evaluation has three parts, namely peak force, the rate of change of the force, and force fatigue (duration of force).

The peak force is the maximum force acting on the trampolinist’s body, which should be within the safety limit threshold. When a force is applied on the trampolinist, it manifests as the trampolinist’s g-force accelerations [39].

The rate of change of force is experienced as shock and impact on the trampolinist’s body. It is represented by jerk [40,41,42], which is also the rate of change of acceleration.

The force fatigue is used to evaluate the extended exposure time effect of trampoline force on the trampolinist. Although the peak force and the rate of force changing are limited in a safe range, the trampoline force could still generate fatigue injury to the trampolinist’s leg joints after a long period of trampolining because, during trampoline performance, the force-bearing parts are focused on the weakest components, namely the back, knees, feet, and long-bones. Thus, in this section, the forces acting on the trampolinist during walking-the-wall and jumping activities are analysed.

### 5.1. Trampolinist Force and Jerk—Walking–the–Wall

When the trampolinist is in contact with the trampoline bed, the back is the force-bearing component of the body.

**Figure 11:**
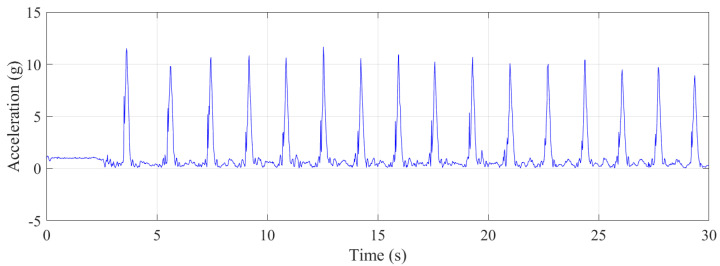
Vector sum of the g-force during the walking–the–wall.

**Figure 12:**
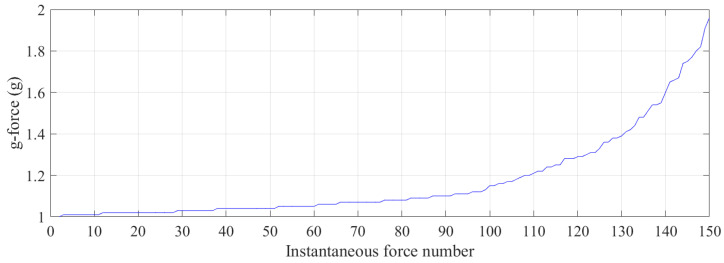
Small variable g-force instances ordered from low to high during the walking-the-wall activity cycles.

As can be seen from Figure 11, initially, the trampolinist experienced a 1 g, force which was due to the reaction force from the ground as the trampolinist was standing on the platform at the top of the wall. While performing on the trampoline, which started at approximately 3.5 s, the trampolinist experienced a very high peak force, with a maximum g-force of approximately 11.5 g due to the trampoline’s reaction force [43] when the trampolinist was in the bottom-dead-centre location, as shown in Figure 3j. This high reaction g-force continued with impact with the trampoline. During walking-the-wall activity cycles, besides the reaction force from the trampoline, other reaction forces arose from the foot contacting the wall, as can be seen from the small variable peak g-forces between the large g-forces. The maximum period for 15 walking-the-wall completed cycles was 2 s; the minimum period was 1.64 s; the average of all the periods was 1.71 s; the standard deviation of all the periods was 0.091 s. Figure 12 illustrates instantaneous g-force instances when the trampolinist’s foot was contacting the wall, ordered from low to high. The g-force from the wall on the trampolinist was more than 1.5 g for 9% of the instances, as can be seen from the graph. For more than 80% of the foot contact with the wall instances, the g-force was greater than Earth’s 1 g. This showed that a relatively higher g-force than the Earth’s gravitational g-force was required for the trampolinist to achieve the walking-the-wall activity. Finally, the g-force experienced was variable. Throughout the activity, it varied from one cycle to the next.

A maximum acceleration of 11.5 g is extreme. It is approximately double what an Air Force Roulette experiences during a flying display [44]. The Pilatas PC-21 plane that the Roulettes fly is the only instrument rated to 8 g and they wear a specially designed compression garment called an anti-g suit to prevent loss of consciousness. However, as the trampolinist experienced these high g-forces momentarily (narrow peaks) and not for extended periods of time, the effect of these g-forces is not comparable to what air force pilots would experience during small-radius turning.

**Figure 13:**
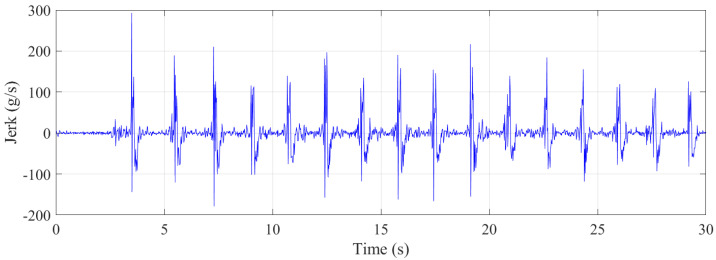
Jerk during the walking–the–wall.

As shown in Figure 13, the maximum jerk experienced by the trampolinist was approximately 200 g/s, which is also extreme. For a lift (elevator) used in a multi-story building, a rate of 2 g/s is considered comfortable, while a rate of 0.7 g/s is used in hospitals, where a high jerk rate may be harmful to patients [45].

### 5.2. Trampolinist Force and Jerk—Jumping

**Figure 14:**
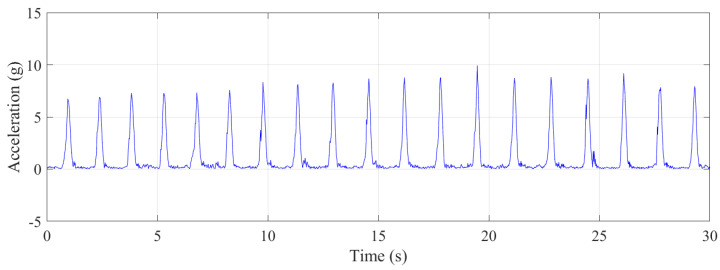
Vector sum of the translational accelerations during the jumping.

**Figure 15:**
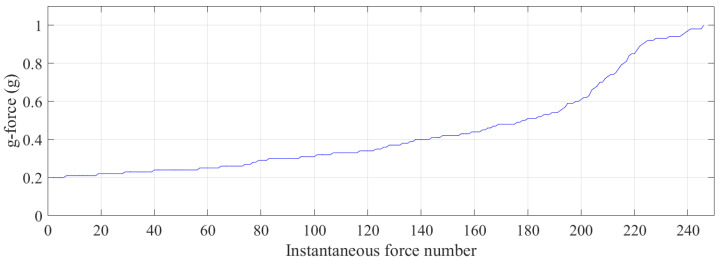
Small variable g-force instances ordered from low to high during the jumping activity cycles.

The g-force experienced during jumping was lower than during the walking-the-wall cycles as the trampolinist’s drop height was lower than during the walking-the-wall activity, as shown in Figure 14. Furthermore, the g-force during jumping activity cycles was more gradual from one cycle to the next. The maximum period for 18 jumping completed cycles was 1.69 s; the minimum period was 1.43 s; the average of all the periods was 1.58 s; the standard deviation of all the periods was 0.085 s. As a result, it can be said that walking-the-wall, on average, takes a slightly longer time to complete a cycle. Figure 15 illustrates the instantaneous g-force when the trampolinist was balancing in the air, after which there was no contact with the trampoline bed. The g-force in the trampolinist’s balancing act was lower than 1.0 g, where, only for 8% of the instances, it was close to 0.9 g for the trampolinist. For more than 50% of the instances, the g-force of the trampolinist was under 0.4 g, indicating a balanced free falling or jumping act that was much lower than the Earth’s gravitational force of 1 g.

**Figure 16:**
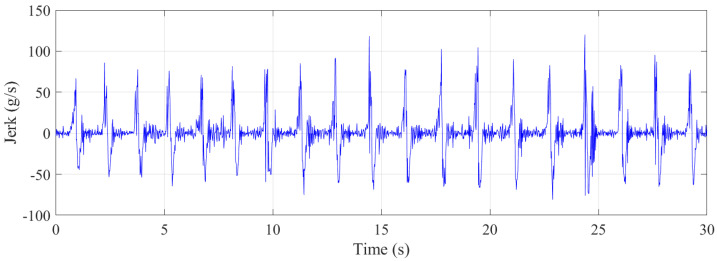
Jerk during the jumping.

Figure 16 depicts the jerk during the jumping. The maximum jerk experienced by the trampolinist was approximately 90 g/s for each part. This rate was much lower than that of the walking-the-wall activity. Thus, it can be said that the walking-the-wall activity cycles were twice as dynamically demanding as the jumping cycles. Although this is the case, human perception of the jerk experience might not function linearly, where doubling the jerk does not necessarily mean doubling of the required fitness level for an activity.

### 5.3. Force and Jerk—Jogging on the Floor

**Figure 17:**
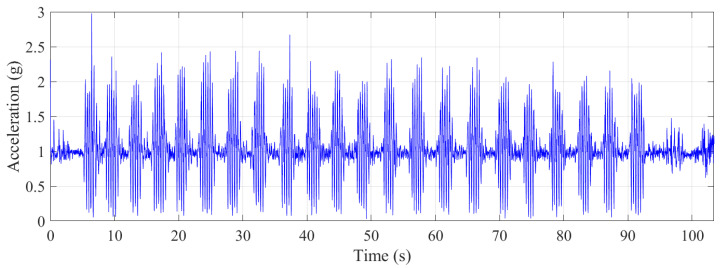
Vector sum of the translational accelerations during jogging on the floor.

**Figure 18:**
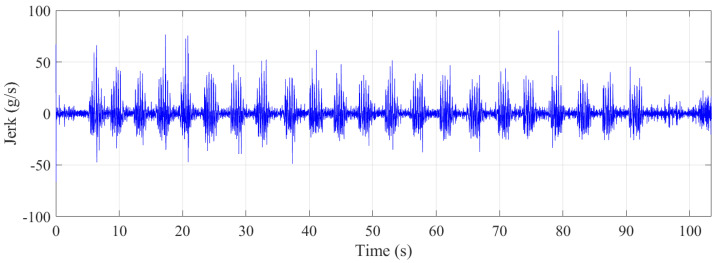
Jerk during jogging on the floor.

For comparison with trampoline walking-the-wall g-force feedback with ground g-force feedback, accelerometer data during normal jogging on the floor were also collected. Figure 17 depicts the vector sum of g-force for jogging on an unpadded floor with more than 20 separate short jogging sessions. The g-force experienced during jogging was much lower than during both jumping and walking-the-wall activities on the trampoline bed. The same can be said for jerk during jogging sessions, as depicted in Figure 18.

**Figure 19:**
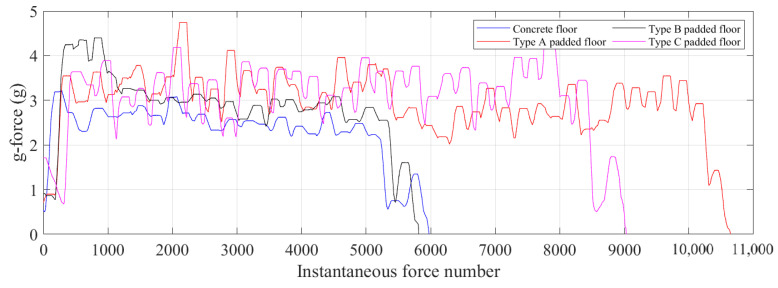
G-force acceleration on a floor with no padding (concrete) and different types of padding applied on the floor.

Furthermore, data were collected for jogging on different floor conditions, both padded and not padded. Figure 19 depicts the instantaneous g-force magnitude experienced through the foot during jogging on different floor conditions. The concrete floor (unpadded) provided the lowest overall g-force feedback compared to a floor that was covered by padding. As can be seen from the plot, Type B padding on the floor increased the overall g-force feedback while jogging for the same duration, roughly 90 s. Type A and C padding was softer than Type B padding, which also increased the g-force feedback through the foot further than without any padding on the concrete floor, even though the jogging lasted for longer than 90 s for these two padding conditions. The introduction of padding on the concrete floor increased the overall g-force by as much as 27%, as can be seen from the graph.

## 6. Walking-the-Wall Stance Time

The stance time has been used by a number of other researchers to better understand biomechanics, particularly the forces within motion. Examples of this include Wilson et al. [46], who studied cheetah hunting dynamics, Hayati et al. [47], who studied canine gait characteristics, Witte et al. [48], who studied the ground reaction force in horses, and Dutto et al. [49], who reported ground reaction forces in horses trotting up an incline and on the level over a range of speeds. As aforementioned, we have previously conducted research on the key techniques in the Korean martial art of taekwondo and observed various transitions between different stances and the stance times [17]. An interesting observation that was consistent with previous sports biomechanics and martial arts literature was the notion of a kinetic chain from the ground to the fist. This essentially refers to the practitioner drawing power from the ground and transitioning it to their fist at the time of impact through body rotation and joint transitions. The importance of stance and stance time has also been emphasised in other popular martial arts, such as boxing [50] and karate [51]. A recent study by [52] of postural stability in athletes investigated the role of age, sex, performance level, and shoe features by evaluating the centre of pressure, sway area, and velocity of the athletes. The results showed that postural stability was mostly influenced by the athlete’s age. It was also observed that long-term use of rigid athletic shoes with stiff ankle support can lead to reduced postural stability. In our future work, it would be interesting to explore similar factors in trampoline athletes and the impact of these factors in performing certain techniques.

From the walking-the-wall activity, it was found that the stance time for a single foot on the wall was nearly half of the stance time when both feet were in contact with the wall while performing a cycle. The stance time for a single foot that was applying pressure on the wall was between 0.25 and 0.40 s. However, the stance time for the walking-the-wall activity can greatly vary; the stance time for jumping on the trampoline bed while applying pressure by the feet was approximately between 0.38 and 0.42 s.

## 7. Conclusions

Safety is of paramount importance during any trampolining activities. In high-performance trampolining activities such as walking-the-wall, accuracy and informed design are critical, as the window for error is very narrow in this manoeuvre.

In this paper, it is predicted that rigid vertical walls would allow the trampolinist to maintain control and retain spatial awareness to a far greater degree than what is achievable with a non-rigid surface on the wall. In the presence of a padded wall, the reaction force from the wall can be considered a variable force that would have higher g-force feedback on the trampolinist’s center of mass to control and manage, as shown by the jogging g-force feedback data on different floor conditions. This study had some limitations, including the use of a single trampolinist. All recordings and measurements were conducted with one specific high-performance athlete. The implication of this is that the outcome will vary slightly for the normal to average performer, or performers of a different weight class. However, the general trend from this research can still persist as g-force feedback demonstrates the overall outcome rather than specifically analysing postural changes with composition. We propose that this limitation does not detract from these findings, as the primary purpose of this study was to assess the ability of trampolinists to maintain control while performing the walking-the-wall manoeuvre on hard compared with impact-attenuating vertical surfaces.

The strength and importance of this study lies in the fact that it offers an overview of the required balance of the trampolinist, with each walk and run up the wall and a controlled fall from height. The findings of our study conclude that unattenuated vertical walls are safer than attenuated vertical walls for walking-the-wall activities within trampoline park facilities as the variable force from the wall has a limited range. Our findings suggest that non-rigid walls can provide an unstable surface with a high degree of force variability from the wall, which would reduce the trampolinist’s control and stability. This may serve to add to, rather than mitigate, the risk involved with this manoeuvre, as control and stability are critical while performing the complex walking-the-wall manoeuvre. It was concluded that applying impact attenuation padding to vertical walls used for walking-the-wall and similar activities increased the likelihood of injury. These findings offer important information to the regulatory bodies providing guidance for the safe installation, structure, and maintenance of indoor trampoline park facilities, in the ongoing effort to develop a robust evidence base for national or international product standards.

## Data Availability

The data presented in this study are available on request from the corresponding author.

## Abbreviations

The following abbreviations are used in this manuscript:

ASD	autism spectrum disorder
HFR	high-frame-rate
IMU	inertial measurement unit
FFT	fast Fourier transform
